# Carbohydrate-binding modules targeting branched polysaccharides: overcoming side-chain recalcitrance in a non-catalytic approach

**DOI:** 10.1186/s40643-021-00381-7

**Published:** 2021-04-12

**Authors:** Jiawen Liu, Di Sun, Jingrong Zhu, Cong Liu, Weijie Liu

**Affiliations:** Jiangsu Key Laboratory of Phylogenomics & Comparative Genomics, School of Life Science, Jiangsu Normal University, No. 101, Shanghai Road, Tongshan New District, Xuzhou, 221116 Jiangsu China

**Keywords:** Carbohydrate-binding module, Protein structure, Protein-carbohydrate recognition, CBM fusion, Hemicellulose, Side-chain recalcitrance, Lignocellulose conversion

## Abstract

Extensive decoration of backbones is a major factor resulting in resistance of enzymatic conversion in hemicellulose and other branched polysaccharides. Employing debranching enzymes is the main strategy to overcome this kind of recalcitrance at present. A carbohydrate-binding module (CBM) is a contiguous amino acid sequence that can promote the binding of enzymes to various carbohydrates, thereby facilitating enzymatic hydrolysis. According to previous studies, CBMs can be classified into four types based on their preference in ligand type, where Type III and IV CBMs prefer to branched polysaccharides than the linear and thus are able to specifically enhance the hydrolysis of substrates containing side chains. With a role in dominating the hydrolysis of branched substrates, Type III and IV CBMs could represent a non-catalytic approach in overcoming side-chain recalcitrance.

## Introduction

Converting lignocellulose into biofuels and other products has attracted tremendous attention and interest (Kubicek and Kubicek 2016; Chandel et al. 2018; Shabih et al. 2018). Utilization of this renewable biomass meets the increasing energy demand and also reduces pollution resulting from improper disposal of agricultural residues (Taha et al. 2016; Rastogi and Shrivastava 2017; Liu et al. 2020). Inefficiency in enzymatic deconstruction of lignocellulose due to its recalcitrance remains one of the major technical bottlenecks for biorefinery despite decades of research (Himmel et al. 2007; Xu et al. 2019). Lignin and hemicellulose are the major factors that contribute to the recalcitrance of lignocellulose and can be removed using various pretreatment processes (Chen et al. 2017; Zoghlami and Paes 2019). However, the removal of hemicellulose is unwieldy and costly because it is also the source of substantial fermentable sugars (Houfani et al. 2020). Therefore, efficient hydrolysis of hemicellulose is a major challenge to the enzymatic conversion of lignocellulose.

Being different from cellulose, hemicellulose is generally branched because it consists of backbone and side chains. Backbone of arabinoxylan, for example, is composed of β-1,4-linked xylose, and the arabinoses linking with xylose unit via α-1,2 or α-1,3 bonds constitute side chains (Chen et al. 2019). Extensive decoration of backbones limiting the accessibility of enzymes is one of the major factors resulting in resistance to enzymatic hydrolysis of hemicellulose (Moreira and Filho 2016; Xin et al. 2016). To overcome the recalcitrance of hemicellulose, some hydrolytic enzymes such as glycosidases and esterases are commonly required to remove the side chains prior to attacking the polysaccharide backbone (Huang et al. 2017; Song et al. 2018; Matsuzawa et al. 2020). However, some special hydrolysis modes also exist. For example, extracellular enzymes from some *Bacteroides* spp. degrade hemicellulose into oligosaccharides with side chains before the debranching step (Rogowski et al. 2015; Déjean et al. 2019). Moreover, certain main-chain degrading enzymes can break backbones efficiently without relying on side-chain acting enzymes. The most common example is xylanases from glycoside hydrolase (GH) family 30, which specifically attack sites of 4-*O*-methylglucuronic acid or arabinose substitution on the xylan backbone without relying on side-chain acting enzymes (St John et al. 2006; Valenzuela et al. 2012; Verma and Goyal 2016). The accommodation of a decorated xylopyranosyl residue in its catalytic cleft eliminates steric clashes of substitution, and the interaction between certain amino acid residues and side chains is a determinant of the specificity of these xylanases (St John et al. 2011; Urbániková et al. 2011). A similar mechanism is employed by some enzymes from other GH families such as *Ct*Xyl5A and *Cj*GH74 to hydrolyze branched substrates (Attia et al. 2016; Labourel et al. 2016). The presence of these enzymes suggests that side-chain-acting enzymes are not always essential to the hydrolysis of branched substrates. Furthermore, the application of this type of backbone enzymes that do not require side-chain acting activity is beneficial to the conversion of branched polysaccharides like hemicellulose. Nevertheless, related reports are still limited.

Carbohydrate-binding modules (CBMs) are a group of non-catalytic domains that can bind to various carbohydrates (Karita 2016). Most CBMs are connected to catalytic domains of carbohydrate-active enzymes via peptide linkers, and only a few of them exist independently. CBMs play critical roles in stimulating enzymatic conversion of lignocellulose and various polysaccharides. The primary mechanism of promoting hydrolysis is increasing enzyme-substrate proximity: CBMs can bring attached catalytic domains to the surface of their substrate, which leads to higher regional enzyme concentration and prolonged contact with substrate (Herve et al. 2010; Orita et al. 2017). Moreover, CBMs are able to maintain the conformation of enzymes, and thus improve thermostability (Meng et al. 2015). In addition, certain CBMs can enhance processivity of enzymes due to boost of binding affinity (Pan et al. 2016). Recent studies also demonstrated that CBMs can confer substrate specificity to catalytic domains by altering binding mode of enzyme (Venditto et al. 2015). With these functions, CBMs have become the most attractive non-catalytic domain in promoting lignocellulose hydrolysis. In recent years, a few CBMs were reported to target polysaccharides containing side chains and thus specifically promote the hydrolysis of branched substrates (Cuskin et al. 2012; Furtado et al. 2018). This meaningful function of CBMs may represent a non-catalytic way to overcome side-chain recalcitrance of hemicellulose and other polysaccharides (describing in “Overcoming side-chain recalcitrance of polysaccharides via CBMs” section). However, reports on this type of CBMs are rare so the role of CBMs in hydrolyzing branched polysaccharides is vague. Here, we review the CBMs that can bind branched polysaccharides and propose a new classification to describe them. CBMs existing in hemicellulases are also summarized based on our proposed classification. Finally, we provide a new conceptual insight about the biological function of CBMs in overcoming side-chain recalcitrance as well as a novel research direction to perfect the role of CBMs in glycan degradation.

## Results and discussion

### A novel classification describing the binding characteristics of CBMs

To date, more than 200,000 CBM sequences are included in the CAZy database (http://www.cazy.org/Carbohydrate-Binding-Modules.html), and these have been classified into 88 families based on amino acid sequence. To describe the functional characteristics of CBMs, another classification based on ligand binding and structural similarity was proposed, in which the CBMs are grouped into three types: Type A CBMs have a flat or platform-like binding site composed of aromatic residues, and they bind the crystalline surfaces of insoluble substrates; Type B CBMs possess a binding site similar to a groove or cleft that can accommodate a single polysaccharide chain; and Type C CBMs lack the extended binding-site grooves of Type B CBMs and thus bind short oligosaccharides, such as monosaccharides, disaccharides, and trisaccharides (Boraston et al. 2004). As the distinction between Type B and C CBMs are sometimes subtle, a modified classification was then proposed, in which Type B and C CBMs are, respectively, redefined as those binding to the interior and termini of glycan chains (Gilbert et al. 2013).

Although the ABC classification provides the structural and functional characteristics of CBMs, diversity of substrates is generally neglected there. Some polysaccharides like glucan and glucomannan are linear while hemicellulose is commonly branched. Although many CBMs can bind both kinds of polysaccharides, the effect of side chains on binding is different (Hernandez-Gomez et al. 2015; Ma et al. 2018; Fredriksen et al. 2019). For example, certain CBMs prefer linear ligands to the branched ones, but some others show specificity for glycan containing side chains (Najmudin et al. 2006; Luis et al. 2013). Those CBMs apparently possess different structures and functions, though they all belong to Type B. To highlight the diversity in binding and function of CBMs, we propose a novel classification to describe CBMs according to their preference in ligand types, in which these are grouped into four types (Table 1). Type I CBMs prefer linear ligands than the branched. Side chains will interfere with the interaction between CBMs and ligands, so affinity of a Type I CBM for branched ligands is much weaker than that for linear ones. A known example of Type I CBMs is *Tm*CBM27 from a mannanase (Man5) of *Thermotoga maritima*, which is able to bind both branched and linear mannan oligosaccharides, but its affinity for G_2_M_5_, a mannopentaose containing two galactosyl side chains, is only 1.1% of that for linear mannopentaose (Boraston et al. 2003). Affinity of Type II CBMs for branched and linear polysaccharides is similar because the steric clashes derived from the side chains are negligible. CBM30 from CelJ of *Clostridium thermocellum*, which exhibits almost equal affinity to β-glucan and xyloglucan, can be defined as a Type II CBM (Najmudin et al. 2006). Type III CBMs not only accommodate branched ligands like Type II CBMs, but also form bonds with side chains of polysaccharides. As side chains positively contribute to binding, Type III CBMs show stronger affinity for branched polysaccharides. CBM65A and CBM65B from endoglucanase *Ec*Cel5A of *Eubacterium cellulosolvens* are Type III CBMs, which engage in hydrophobic interactions with the xylose side chains and thus display tenfold greater affinity for xyloglucans than linear β-glucans (Luis et al. 2013). Type IV CBMs mainly bind to the side chains but not the polysaccharide backbones, thereby showing specificity for branched polysaccharides as well. An example of Type IV CBMs is *Ct*CBM35-Gal, which targets α-1,6-galactose residues of galactomannan (Correia et al. 2010). As affinity of a CBM for linear and branched polysaccharide cannot be exactly identical, the distinction between Type II and Type I or Type III CBMs is sometimes ambiguity. To distinguish Type II CBM from the other two, we propose to define the CBMs whose affinity for linear and branched ligands differs by less than 100% as the Type II.

**Table 1:** Available information of CBMs involved in the proposed four-type classification

Name	Modular architecture	Enzyme type	Ligands	Specificity	ABC classification	Four-type classification	References
*Tm*CBM27	GH5-CBM27	Mannanase	Mannan	Linear	Type B	Type I	Boraston et al. (2003)
*Cc*CBM17	GH5-CBM17	Cellulase	β-Glucans/xyloglucan	Linear	Type B	Type I	Najmudin et al. (2006)
*B*spCBM28	GH5-CBM17-CBM28	Cellulase	β-Glucans/xyloglucan	Linear	Type B	Type I	Najmudin et al. (2006)
CBM79-1_RfGH9_	GH9-CBM79-CBM79	Cellulase	β-Glucans/xyloglucan	Linear	Type B	Type I	Venditto et al. (2016)
CBM48	CBM41-CBM48-GH13	Pullulanase	Amylose/amylopectin	Linear	Type B	Type I	Hedin et al. (2017)
*Rt*CBM11	GH26-GH5-CBM11	Cellulase	β-Glucans/xyloglucan	Linear	Type B	Type I	Furtado et al. (2018)
Man5C-CBM35	CBM5-CBM10-CBM35-GH5	Mannanase	Mannan	Similar^a^	Type B	Type II	Bolam et al. (2004)
*So*CBM13	GH10-CBM13	Xylanase	Xylan	Similar^b^	Type B	Type II	Fujimoto et al. (2004)
CBM30, CBM44	CBM30-GH9-GH44-CBM44	Cellulase	β-Glucan/xyloglucan	Similar	Type B	Type II	Najmudin et al. (2006)
*Cf*CBM4-1	CBM4-CBM4-GH9	Cellulase	β-Glucan/xyloglucan	Similar	Type B	Type II	Najmudin et al. (2006)
*Pe*CBM29	Cellulosome	Cellulosome	β-Glucans/xyloglucan	Branched	Type B	Type III	Najmudin et al. (2006)
Unnamed	GH30-CBM35-CBM6	Xylanase	Methylglucuronoxylan	Branched^b^	Type B	Type III	St John et al. (2011)
X-2 L110F	CBM4-CBM4-GH10	Xylanase	β-Glucans/xyloglucan	Branched	Type B	Type III	von Schantz et al. (2012)
CBM65A, CBM65B	CBM65-GH5-CBM65-GH5	Cellulase	β-Glucan/xyloglucan	Branched	Type B	Type III	Luis et al. (2013)
CBM75_RfGH43_	GH43-CBM75	Arabinofuranosidase	β-Glucans/xyloglucan	Branched	Type B	Type III	Venditto et al. (2016)
CBM76_RfGH44_	GH44-CBM76	Cellulase	β-Glucans/xyloglucan	Branched	Type B	Type III	Venditto et al. (2016)
CBM78_RfGH5_	GH5-CBM78	Cellulase	β-Glucans/xyloglucan	Branched	Type B	Type III	Venditto et al. (2016)
CBM80_RfGH5-1/2_	GH5-CBM80-GH5	Cellulase/mannanase	β-Glucans/xyloglucan	Branched	Type B	Type III	Venditto et al. (2016)
*St*CBM64C	GH10-CBM64	Xylanase	β-Glucans/xyloglucan	Branched	Type A	Type III	Pires et al. (2017)
Stbd1CBM	CBM20	Human starch-binding domain containing protein 1	Amylopectin/amylose	Branched	Type B	Type III	Skurat et al. (2017)
*Rt*CBM11mut	CBM11-GH12	Xyloglucanase	β-Glucans/xyloglucan	Branched	Type B	Type III	Furtado et al. (2018)
*Ak*CBM42	GH54-CBM42	Arabinofuranosidase	Arabinofuranose	Branched	Type C	Type IV	Miyanaga et al. (2006)
Xyl-CBM35	GH10-CBM35	Xylanase	Glucuronic acid	Branched	Type C	Type IV	Montanier et al. (2009)
*Ct*CBM35-Gal	GH39-CBM35-CBM35	Unknown	α-d-galactose	Branched	Type C	Type IV	Correia et al. (2010)
*Bs*CBM66	GH32-CBM66	β-Fructosidase	Fructosides	Branched	Type C	Type IV	Cuskin et al. (2012)
Laminin_G_3	CBM42-GH43- CBM42-GH43-GH43	Arabinofuranosidase	Arabinooligosaccharide	Branched	Type C	Type IV	Sakka et al. (2019)

^a^Similar indicates that affinity for linear and branched ligands differs by less than 100%

^b^Specificity is inferred based on its binding characteristics and structural information

**Fig. 1:**
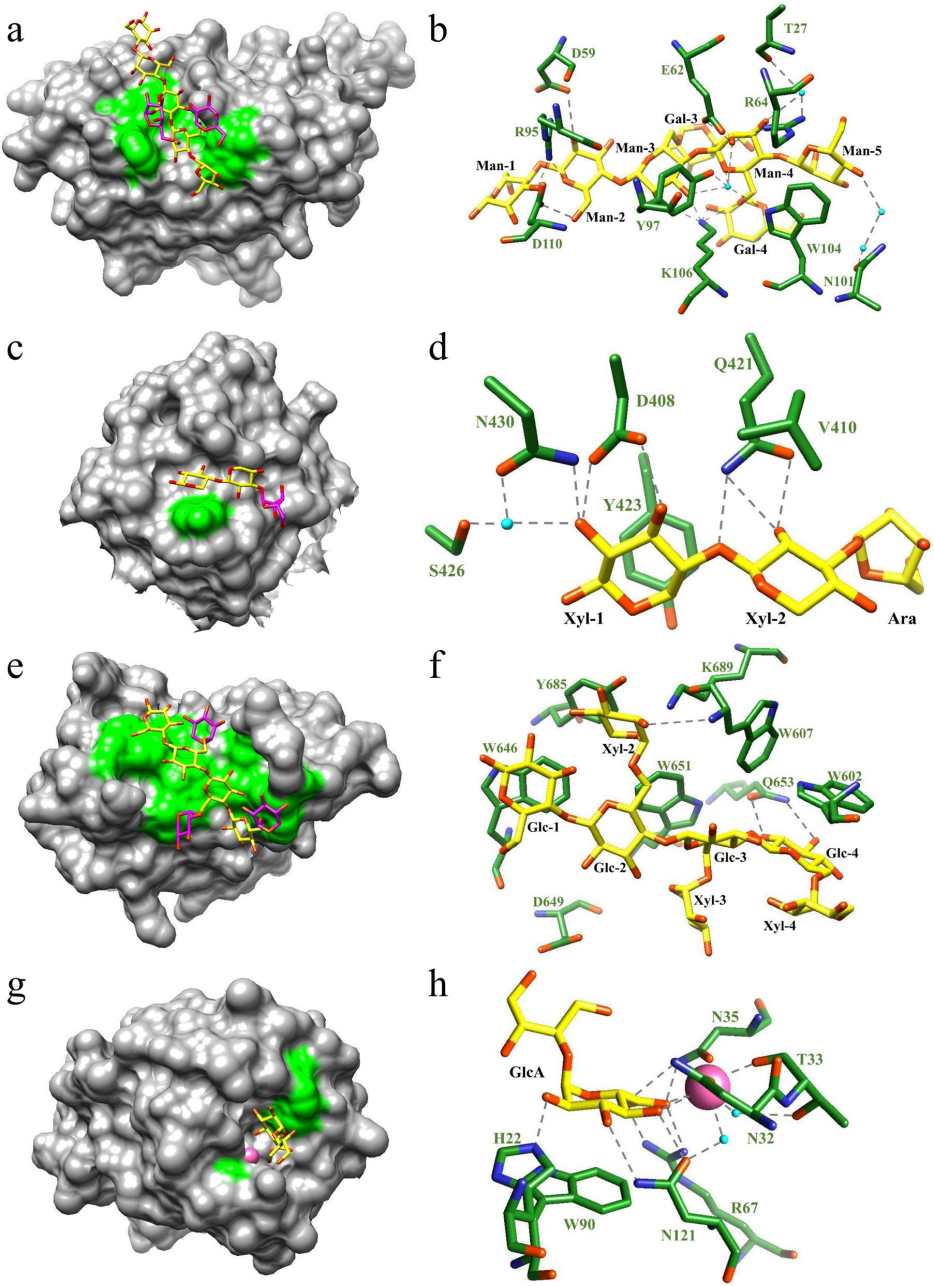
Structural features of CBMs binding to branched ligands. **a** *Tm*CBM27-G_2_M_5_ complex, **c** *So*CBM13-Ara*f*X2 complex, **e** CBM65B-XXXG complex, and **g** Xyl-CBM35 in complex with a disaccharide containing glucuronic acid are displayed in a solvent-accessible surface format. Ligand binding sites of **b** *Tm*CBM27, **d** *So*CBM13, **f** CBM65B and **h** Xyl-CBM35 are also shown (Boraston et al. 2003; Fujimoto et al. 2004; Montanier et al. 2009; Luis et al. 2013). *So*CBM13-Ara*f*X3 complex is not shown here. Aromatic residues involving in ligand binding are shown in green. Pink and cyan balls indicate Ca^2+^ and water molecules, respectively

Effect of side chains on the affinity of CBMs for ligands is decisive factor in such classification, which is essentially determined by shape of CBM binding site and interaction between side chains and amino acid residues. Binding sites of Type I CBMs are too narrow to accommodate the side chains of branched polysaccharides. For example, a galactose substitution of G_2_M_5_ cannot be accommodate in the subsite 4 of *Tm*CBM27, and the interaction between the other substitution of G_2_M_5_ and Trp28 weakens the affinity of *Tm*CBM27 for this ligand (Fig. 1a, b). The whole or part of binding sites of Type II CBMs is more extended and thus can accommodate side chains of branched ligands. *So*CBM13 is the sole Type II CBM whose structure was solved in complex with branched ligands. *So*CBM13 makes hydrogen bonds only with xylose units when binding with 2(2)-α-l-arabinofuranosyl-xylobiose (Ara*f*X2) or 3(2)-α-l-arabinofuranosyl-xylotriose (Ara*f*X3), while it makes no direct contact with the arabinose substitutions in both of branched oligosaccharides. Therefore, effect of side chains on the interaction between *So*CBM13 and ligands is insignificant (Fig. 1c, d). Type III CBMs also possess extended binding clefts. In addition, their amino acid residues can make bonds with side chains, which enhances the affinity for branched ligands. The xylose side chains of XXXG, for instance, can be accommodated unperturbedly in the cleft-like binding site of the Type III CBM65B. Moreover, the Xyl-2 and Xyl-3 substitutions of XXXG make hydrophobic interactions and apolar contacts with tryptophans of CBM65B, respectively, which contribute significantly to CBM65B recognition (Fig. 1e, f). Type IV CBMs primarily bind with side chains but not the backbone and accordingly, their binding-site grooves are not as extended as those of other three types of CBMs. Inspection of the structure of a Type IV CBM, for example the Xyl-CBM35, is able to identify a shallow indentation-like binding site that can accommodate a single monosaccharide. Xyl-CBM35 recognizes and establishes bonds with the glucuronic acid ligand, which is also the side-chain of glucuronoxylan (Fig. 1g, h).

Our proposed novel classification mainly describes those CBMs that bind to soluble polysaccharides or oligosaccharides, which means Type A CBMs are generally excluded. An exceptive example is *St*CBM64C, which is a Type A CBM, that binds xyloglucan with a significantly stronger affinity than β-glucan (Pires et al. 2017). In spite of covering limited CBMs, this four-type classification displays more detailed binding characteristics than the ABC classification. For example, the specificity of CBM65A from *Ec*Cel5A for xyloglucan instead of glucan is clearly depicted when the CBM is grouped into Type III, but this preference for branched polysaccharides is not indicated in the ABC classification. Another significant advantage of the four-type classification is indication of appropriate type of CBMs to promote enzymatic conversion of polysaccharides with different structures. It was reported that, for instance, Type I *Rt*CBM11 cannot promote catalytic efficiency of XegA for xyloglucan but Type III *Rt*CBM11mut can, although they both belong to Type B (Furtado et al. 2018). The other case is Type III CBM44 which stimulates enzyme activity for xyloglucan more than that for carboxymethylcellulose by targeting effect, suggesting that promotion of CBMs on activities is influenced by their ligand specificity (Najmudin et al. 2006).

### CBMs targeting decorated hemicellulose

Hemicellulose is the most abundant branched polysaccharide with diverse structures, and is resistant to hydrolysis because of containing side chains (Moreira and Filho 2008; Moreira and Filho 2016; Xin et al. 2016). Among hemicelluloses, xylan, xyloglucan, and mannan are very common, whereas β-1,3/1,4-glucan and galactan are less abundant (Table 2) (Alvarez et al. 2016; Zhou et al. 2017; Naidu et al. 2018; Singh et al. 2018). CBMs targeting xylan, xyloglucan and mannan from each type are summarized below, respectively.

**Table 2:** Main types of polysaccharides present in hemicellulose (Alvarez et al. 2016; Zhou et al. 2017; Naidu et al. 2018; Singh et al. 2018)

Type	Backbone	Side chain^a^	Biological origin
Arabinoxylan	β-1,4-Xyl	ɑ-(1,2)/(1,3)-Ara	Herbage
Glucuronoxylan	β-1,4-Xyl	ɑ-(1,2)-GlcA*O*Me	Hardwood
Arabinoglucuronoxylan	β-1,4-Xyl	ɑ-(1,2)/(1,3)-Ara and ɑ-(1,2)-GlcAOMe	Herbage
Xyloglucan	β-1,4-Glc	ɑ-(1,6)-Xyl, ɑ-(1,2)-Fuc, ɑ-(1,3)-Gal, et al	Extensive
Glucomannan	β-1,4-Man/Glc	Rare	Softwood
Galactoglucomannan	β-1,4-Man/Glc	ɑ-(1,6)-Gal	Softwood

^a^This column only displays the carbohydrate side chains of hemicellulose

*So*CBM13 is the first reported CBM that can accommodate branched xylan (Fujimoto et al. 2004). However, *So*CBM13 does not directly interact with either 4-*O*-methyl-α-d-glucuronosyl or arabinofuranosyl side-chain and thus is probably a Type II CBM. In 2006, the first Type IV CBM targeting arabinofuranosyl residues of arabinoxylan, *Ak*CBM42, was reported (Miyanaga et al. 2006). It is also the first CBM that recognizes the side-chain monosaccharides of branched hemicellulose. Subsequently, three Type IV CBMs binding glucuronic acid were found, which may play a role in targeting glucuronoxylan (Montanier et al. 2009). The Type III CBM binding branched xylans was the last reported: CBM from XynC preferentially interacts with 4-*O*-methylglucuronoxylan (St John et al. 2011).

Type I, Type II and Type III CBMs that can accommodate branched xyloglucans were all first reported in 2006: *Ct*CBM11, *Cc*CBM17 and *B*spCBM28 show considerably stronger affinity to cellohexaose than xyloglucan, which belong to Type I; CBM44 and CBM30 from *Ct*Cel9D-Cel44A display similar affinity to xyloglucan and undecorated β-glucans and thus could be classified as Type II; *Pe*CBM29-2 shows stronger affinity to xyloglucan than cello-oligosaccharide, suggesting that it is a Type III CBM (Najmudin et al. 2006). In 2012, an engineered CBM (X-2 L110F) was reported to display approximately tenfold stronger affinity to xyloglucans than β-glucans, which is a canonical characteristic of the Type III (von Schantz et al. 2012). Thereafter, more unengineered Type III CBMs were identified (Luis et al. 2013; Venditto et al. 2016).

In the case of mannan, the first CBM binding with branched ligand, namely, *Tm*CBM27, was reported in 2003 and was classified as the Type I (Boraston et al. 2003). Type II Man5C-CBM35 was subsequently found, which displays similar affinity to branched and linear mannans (Bolam et al. 2004; Tunnicliffe et al. 2005). Type IV *Ct*CBM35-Gal that binds to d-galactose as well as α-galactose residues in galactomannan was reported in 2010 (Correia et al. 2010). Interestingly, the catalytic domain linking with *Ct*CBM35-Gal is not a mannanase, so the enzyme containing Type IV CBMs and meanwhile attacking mannan backbones, has not been identified to date.

Numerous Type I CBMs that target linear polysaccharides such as soluble cellulose and glucomannan have been identified. In contrast, CBMs binding to branched polysaccharides are not easily grouped because distinction between the Type II, III and IV lack of concern. For example, Type III CBMs have not been found in mannanases, and the xyloglucanase containing Type IV CBM has not been reported as well. Limited reports are mainly attributed to the shortage of quantitative analysis of affinity for both linear and branched ligands to CBMs via, for example, isothermal titration calorimetry or microscale thermophoresis. Indeed, the functions of CBMs have been extensively investigated, but most studies focus on either linear or branched ligands. Typical commercial soluble polysaccharides such as arabinoxylan, glucuronoxylan and galactomannan are branched, and linear oligosaccharides are contrary more common than the branched ones. Disproportionate use of linear and branched ligands results in difficulty of classifying CBMs into the four types. Xyloglucan is exceptive because its unbranched form, namely β-glucan, is very common. Affinity researches using both xyloglucan and β-glucan are extensive, and thus more than 60% of classified CBMs are of xyloglucan binding capacity (Table 1).

### Overcoming side-chain recalcitrance of polysaccharides via CBMs

**Fig. 2:**
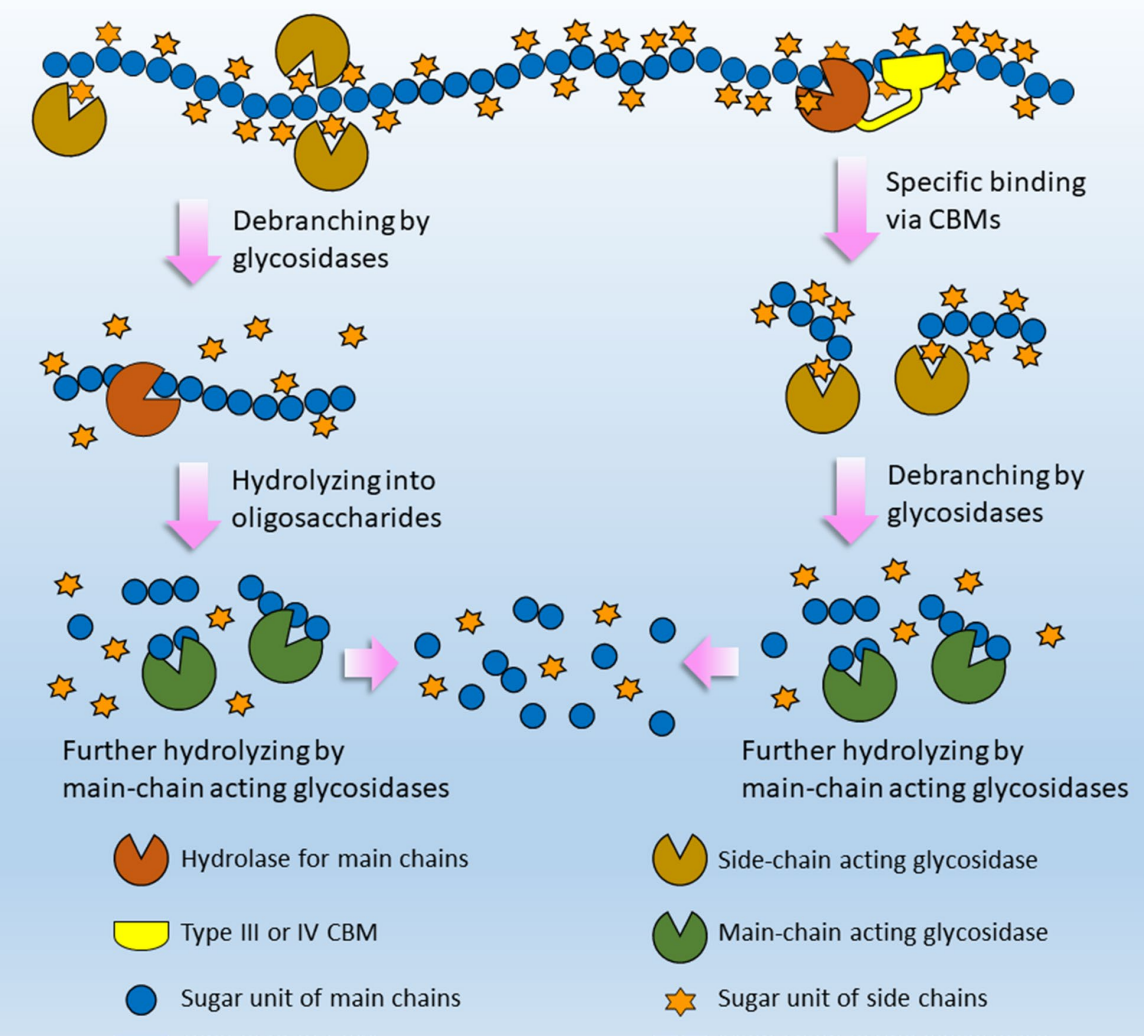
Model of two approaches to overcome side-chain recalcitrance dominated by debranching enzymes and CBMs, respectively. Side chains are removed by debranching enzymes prior to attacking the polysaccharide backbone in the canonical approach. With the assistance of Type III and IV CBMs, enzymes like hemicellulases that act on backbones are able to effectively and directly hydrolyze main chains in the presence of side chains, which means the recalcitrance is overcome or weakened. In both approaches, hydrolysis of main chains and side chains could be simultaneous, but it is displayed successively for better comparison between the two approaches

Activity of catalytic domain toward different substrates is affected, or even determined by ligand type of attached CBM. A recent study has shown that hydrolytic activities of multifunctional CelE toward cellulose, lichenan, xylan and mannan increased by more than 200% when the catalytic domain was recombinantly fused with different CBMs having corresponding binding specificities (Walker et al. 2015). Another example also demonstrated that catalytic activities of multifunctional endoglucanase, Cel5E, toward oat-spelt xylan, ball-milled cellulose and microcrystalline cellulose increased by 150%, 900% and 200% when fused with CBM6, CBM11 and CBM3, respectively, which target corresponding substrates (Ichikawa et al. 2016). As Type III and IV CBMs prefer to bind branched substrates, fusion of those CBMs to main-chain degrading enzymes is probably able to dominate the degradation of decorated polysaccharides directly. There are a few cases supporting our point so far. It was reported that *Rt*CBM11 displayed stronger affinity for linear polysaccharides than for xyloglucan, and a mutant (Type III) derived from *Rt*CBM11 showed inverse substrate specificity that prefers xyloglucans (Furtado et al. 2018). Chimeric enzymes composed of mutational *Rt*CBM11 and XegA, a GH12 xyloglucanase, displayed 38% higher *K*_cat_/*K*_m_ on xyloglucan than that for XegA. In contrast, the wild-type *Rt*CBM11 only increased the *K*_cat_/*K*_m_ by 9% when fused to XegA. The other known example is the *Bs*CBM66 of *exo*-acting β-fructosidase SacC, which binds to the terminal fructose of fructans (Type IV) (Cuskin et al. 2012). Deletion of *Bs*CBM66 from SacC resulted in a 100-fold reduction in catalytic efficiency on fructan containing side chains (levan) but only led to a 32% lower activity against the linear one (inulin). Moreover, appending *Bs*CBM66 to another β-fructosidase from *Bacteroides thetaiotaomicron* resulted in a 120-fold increase in activity against levans and only had negligible influence on the hydrolysis of inulin. To sum up, both Type III and IV CBMs are of specific stimulation for hydrolysis of branched substrates. Commonly, some auxiliary enzymes like glycosidases are commonly necessary to enhance the hydrolysis of polysaccharide backbone by removing side chains to reduce recalcitrance. Type III and IV CBMs, however, can also dominate enzymatic conversion of branched substrates by specific binding to the decorated fraction. Being different from the catalytic way represented by glycosidases, CBMs could be a non-catalytic way of overcoming side-chain recalcitrance (Fig. 2).

Type III and IV CBMs would be crucial and very helpful to the enzymatic conversion of hemicellulose because those polysaccharides are generally branched. Although most hemicellulases do not possess Type III or IV CBMs, those CBMs can be recombinantly fused with catalytic domains of some crucial enzymes to enhance hydrolysis of branched hemicellulose using protein engineering technology (Maharjan et al. 2018). However, there are some issues to resolve. The first problem, as well as the most serious one, is that role of Type III and IV CBMs in hydrolysis of branched substrates remains to be confirmed by more investigations. Lack of relative studies results from, on one hand, scarcity of identified Type III and IV CBMs, and on the other hand, uneven use of linear and branched ligands in enzymology researches (Morrill et al. 2015; Levi Hevroni et al. 2020). Secondly, conditions that CBMs take effect are vague. For example, *K*_m_ of a mannanase (SACTE_2347) for branched galactomannan may not decrease more than that for linear mannan, when a Type IV CBM was attached (Takasuka et al. 2014). Roles of CBMs could be influenced by characteristic of catalytic domains, quantity of CBMs or other factors, which needs more researches to be revealed (Yi et al. 2013; Mollerup et al. 2016). Thirdly, feasibility of applying Type III and IV CBMs to industrial enzymolysis remains to be analysed. Branched oligosaccharides are one of the main products in hydrolysis by main-chain degrading enzymes. For a higher yield of fermentable monosaccharides, a handful of side-chain acting enzymes are still needed. The efficiency and cost of employing hemicellulases containing Type III and IV CBMs should be evaluated and compared with those of using additional auxiliary enzymes.

## Conclusions

CBMs binding to soluble polysaccharides or oligosaccharides can be grouped into four types based on their preference in ligand types. Type III and IV CBMs preferentially bind to branched ligands rather than the linear, thereby facilitating the hydrolysis of polysaccharides that contain side chains. Being different from the catalytic way that employs debranched enzymes, CBMs could represent a non-catalytic approach of overcoming side-chain recalcitrance by specific binding. Application of these natural or fused enzymes containing Type III or IV CBMs would help to enzymatic conversion of decorated hemicellulose in biofuel, food and prebiotics industries. However, numerous CBMs remain to be classified via quantitative analysis of affinity for both branched and linear ligands. Moreover, feasibility of employing Type III and IV CBMs to deal with branched substrates requires additional investigation, because role of CBMs is affected by many factors. In addition, the efficiency and cost of using hemicellulases with Type III and IV CBMs in industrial process need to be evaluated. In summary, the roles of CBM in stimulating degradation of branched polysaccharides are just preliminary and deficient, based on current studies. It is undisputed, however, that this novel biological function of CBMs will be revealed by further researches.

## Data Availability

Data sharing is not applicable to this article as no datasets were generated or analysed during the current study.

## Abbreviations

Ara*f*X2: 2(2)-α-L-Arabinofuranosyl-xylobiose
Ara*f*X3: 3(2)-α-L-Arabinofuranosyl-xylotriose
CBM: Carbohydrate-binding module
GH: Glycoside hydrolase
